# Long noncoding RNA NEAT1 regulates radio-sensitivity via microRNA-27b-3p in gastric cancer

**DOI:** 10.1186/s12935-020-01655-4

**Published:** 2020-12-03

**Authors:** Ying Jiang, Shan Jin, Shisheng Tan, Yingbo Xue, Xue Cao

**Affiliations:** Department of Oncology, The People’s Hospital of Guizhou, No. 83, Zhongshan East Road, Guiyang, 550002 Guizhou China

**Keywords:** Gastric cancer, NEAT1, miR-27b-3p, Radio-sensitivity

## Abstract

**Background:**

Long noncoding RNA nuclear-enriched abundant transcript 1 (NEAT1) exhibits an oncogenic role in multiple cancers, including gastric cancer (GC). But, the functions of NEAT1 in modulating radio-sensitivity of GC and its potential molecular mechanisms have not been totally elucidated.

**Methods:**

qRT-PCR was performed to detect the expressions of NEAT1 and microRNA-27b-3p (miR-27b-3p). Kaplan–Meier survival curves for NEAT1 expression in GC created using KM Plotter. Colony formation assay was used to determine the survival fraction. Cell apoptosis was evaluated by flow cytometry. Luciferase reporter assay was used to verify the relationship between miR-27b-3p and NEAT1.

**Results:**

NEAT1 was highly expressed while miR-27b-3p was downregulated in GC tissues and cells. NEAT1 was negatively correlated with that of miR-27b-3p and associated with poor overall survival. Moreover, NEAT1 and miR-27b-3p varied inversely after radiation in GC tissues and cells. Loss of NEAT1 or upregulation of miR-27b-3p increased the effect of radiation on cell survival fraction inhibition and apoptosis promotion. In addition, NEAT1 negatively regulated the expression of miR-27b-3p in GC cells. Interestingly, the depletion of miR-27b-3p dramatically attenuated the NEAT1 knockdown-mediated function in AGS and MKN-45 cells treated with radiation in vitro. Similarly, downregulation of NEAT1 enhanced the radiation-mediated inhibition of tumor growth, which was mitigated by decrease of miR-27b-3p.

**Conclusion:**

NEAT1 depletion enhanced radio-sensitivity of GC by negatively regulating miR-27b-3p in vitro and in vivo.

## Background

Gastric cancer (GC) is the most common malignant tumors in the digestive system and the leading cause of cancer mortality worldwide [1–3]. It has been reported that approximately 990,000 people are diagnosed with GC worldwide every year, of whom about 738,000 die from this disease [4]. Although researchers have made great progress in anti-cancer treatment during the last decades, the overall survival of GC is still low. Radiotherapy has been the primary method for GC [5, 6], whereas the radio-resistance is an obstacle of radiotherapy. Therefore, it has great significance to explore the exact mechanisms underlying the radiation response of GC.

It is well documented that long noncoding RNAs (lncRNAs) are crucial in regulating cellular processes such as proliferation, metastasis, and apoptosis [7, 8]. Moreover, the exploration of lncRNAs in cancers has been reported [9]. For example, lncRNA Colorectal Neoplasia Differentially Expressed (CRNDE) is upregulated in colorectal cancer (CRC) tissues and the depletion of CRNDE notably represses proliferation while enhances apoptosis of CRC cells both in vitro and in vivo [10]. Nie et al. [11] reported that zinc finger NFX1-type containing 1 antisense RNA 1 (ZFAS1) expression is overexpressed in GC and is associated with poor prognosis and shorter survival. Meanwhile, decrease of ZFAS1 impeded GC cells proliferation and induces apoptosis in vitro. Therefore, we thought that identification the function of more lncRNAs in GC may help us understand the molecular mechanisms of GC progression. Furthermore, many studies have showed involvement of lncRNAs in radio-resistance of various cancers. A previous study revealed that taurine-upregulated gene 1 (TUG1) is required for the cell metastasis of human bladder cancer [12]. They further disclosed that TUG1 promotes radio-resistance through miR-145/ZEB2 axis. MALAT1 (metastasis-associated lung adenocarcinoma transcript 1) was recently shown to be upregulated in nasopharyngeal carcinoma (NPC) cells and tissues [13]. Besides, MALAT1 knockdown increases the sensitization of NPC cells to radiation. Those data lncRNAs may be responsible for the modulation of radiotherapy of cancers. Recently, lncRNA nuclear-enriched abundant transcript 1 (NEAT1) was reported to be overexpressed in GC cell lines and tissues [14]. Moreover, they also provided the evidence that the high level of NEAT1 is associated with clinical stage, histological type, lymph node metastasis, and distant metastasis of GC. Silencing of NEAT1 induces cell metastasis of GC. Despite the function of NEAT1 in GC has been investigated, the role of NEAT1 in sensitivity of GC response to radiation remains dismal. MicroRNAs (miRNAs) are widely dysregulated in various cancers and participates in the development of cancer by interacting with lncRNAs [8, 13]. Previous study recently demonstrated that miR-27b-3p is lower-expressed in GC clinical samples and upregulation of miR-27b-3p suppresses cell proliferation and xenograft tumors in vivo [15]. However, the role of miR-27b-3p in radio-sensitivity of GC is still on the brink of realization.

Therefore, this study aimed to the role of lncRNA NEAT1 in process of radiation response of GC in vitro and in vivo and its correlation with miR-27b-3p.

## Materials and methods

### Clinical samples

The gastric tumor tissues and adjacent normal tissues were obtained from thirty-one pairs of GC patients which had not received chemo- or radio-therapy in The People’s Hospital of Guizhou. Moreover, another ten GC patients were also enrolled to analyze the expression changes of NEAT1 and miR-27b-3p before and after radiation treatment. All patients had signed informed consent. The experiments are approved by the Ethics Committee of Ethics Committee of The People’S Hospital of Guizhou. Table 1 presents the Demographic characteristics and clinicopathologic features of gastric cancer patients.

**Table 1:** Demographic characteristics and clinicopathologic features of gastric cancer patients (n = 41)

Parameter	Case	Expression of NEAT1		*P* value
		Lower (n = 17)	Higher (n = 24)	
Age (years)				
≤ 60	24	8	16	0.3349
> 60	17	9	8	
Sex				
Male	21	7	14	0.3499
Female	20	10	10	
Lymphatic metastasis				
No	17	11	6	0.0230*
Yes	24	6	18	
TNM stage				
I + II	19	12	7	0.0124*
III	22	5	17	

*P < 0.05, Fisher’s excat test

### Cell transfection and radiation treatment

Human gastric epithelium cell GES-1 and GC cell lines (NCI-N87, SGC-7901, MKN-45, and AGS) were obtained from American Type Culture Collection (Manassas, VA, USA). Cells were cultured in Dulbecco’s Modified Eagle’s Medium (DMEM; Thermo Fisher Scientific, Waltham, MA, USA) supplemented with 20% fetal bovine serum (Grand Island, NY, USA) and 1% penicillin/streptomycin (Sigma, St. Louis, MO, USA) solution, in a humidified atmosphere containing 5% CO_2_ at 37 °C. MKN-45 and AGS cells were irradiated with 4 Gy X-radiation and collected every 2 h within 24 h post radiation. Small interfering RNA for NEAT1 (shNEAT1#1, shNEAT1#2, and shNEAT1#3), NEAT1 overexpression plasmid (NEAT1), pcDNA 3.0 vector (vector), miR-27b-3p mimic (miR-27b-3p), mimic negative control (miR-NC), miR-27b-3p inhibitor (anti-miR-27b-3p), inhibitor negative control (anti-miR-NC) were transfected into MKN-45 and AGS cells using lipofectamine 3000 (Thermo Fisher Scientific) before 4 Gy exposure. The shRNA, miRNA mimic and miRNA inhibitor sequences are shown in Additional file 1: Table S1.

### qRT-PCR assay

Trizol reagent (Invitrogen, Carlsbad, CA, USA) was used to extract total RNAs from cells or tissues. Then, RNAs were reversely transcribed into complementary DNA using TaqMan® MicroRNA Reverse Transcription kit (Biosystems, Forster City, CA, USA). PCR was performed using PerfeCTa SYBR Green QPCR Fast Mix ROX (Quanta Bioscience, Gaithersburg, MD, USA). NEAT1 forward: 5′-GTACGCGGGCAGACTAACAC-3′; NEAT1 reverse: 5′- TGCGTCTAGACACCACAACC-3′; GAPDH forward: 5′-AGAAGGCTGGGGCTCATTTG; GAPDH reverse: AGGGGCCATCCACAGTCTTC; miR-27b-3p forward: 5′-CGCGTTCACAGTGGCTAAG-3′, miR-27b-3p reverse: 5′-GTGCAGGGTCCGAGGT-3′; U6 forward, 5′-CTCGCTTCGGCAGCACA-3′, U6 reverse, 5′-AACGCTTCACGAATTTGCGT-3’.

### Colony formation assay

The survival fraction was determined using colony formation assays. The transfected cells were irradiated with 0, 2, 4, 6, 8 Gy X-radiation and incubated for another 12 days. The colonies were fixed with 6% paraformaldehyde (Sigma) and stained with 0.5% crystal violet (Sigma). The numbers of colonies (> 50 cells) were counted in five randomly chosen fields. The survival fraction was calculated as: (number of colonies/number of cells plated)_irradiated_/(number of colonies/number of cells plated)_non-irradiated_. Each group was conducted with three replicates.

### Cell apoptosis assay

Cells were introduced with above mentioned plasmids or oligos and then were treated with a 4 Gy radiation at 24 h post transfection. Cell apoptosis was determined using FITC Annexin V Apoptosis Detection Kit (BD Biosciences, Franklin Lakes, NJ, USA) according to the manufacturer’s instructions. Cell apoptotic rate was detected by a FACS Cali bur flow cytometer (BD Biosciences).

### Luciferase reporter assay

The potential binding sites of NEAT1 in miR-27b-3p were predicted by Starbase software. The mutant type (NEAT1-Mut) was made with GeneArt™ Site-Directed Mutagenesis PLUS System (Thermo Fisher Scientific). The sequence of NEAT1 contained wild type or mutant type binding sites was synthesized by PCR and inserted into pMIR-REPORT™ (Thermo Fisher Scientific) to construct NEAT1 wild type reporter gene plasmid (NEAT1-Wt) or NEAT1 mutant type reporter gene plasmid (NEAT1-Mut). NEAT1-Wt or NEAT1-Mut and miR-NC, miR-27b-3p mimic, miR-27b-3p inhibitor or anti-miR-NC were transfected into MKN-45 and AGS cells using lipofectamine 3000 (Thermo Fisher Scientific). The luciferase activity was evaluated using the Dual Luciferase Reporter Assay System (Promega, Madison, WI, USA).

### Stable cell line production

For construction of NEAT1 stable knockdown MKN-45 cells, and NEAT1 stable knockdown MKN-45 cells with miR-27b-3p stable knockdown, pEZX-AM04p vector was used to construct the lentiviral vectors (GeneCopoeia, Guangzhou, China). Following the manufacturer’s protocol, 10 μg lentiviral vectors and lentiviral packaging plasmids were transfected into the HEK293T cells. 72 h upon transfection, the cell culture supernate was collected separately, and then added into the MKN-45 cells within logarithmic growth phase at the density of 1 × 10^6^ per well. After transfection for 24 h, puromycin (1 μg/ml) was added to the culture medium to select positively transduced cells. After 4 times of passages in selection medium, the transfection effects were confirmed by qRT-PCR.

### In vivo experiments

The experiments were approved by the animal care and experiments committee of The People’S Hospital of Guizhou. The 24 female BALB/c nude mice (20–22 g, 4–6 weeks) were purchased from Shanghai Laboratory Animal Center (SLAC). Mice were injected with 5 × 10^6^ MKN-45 cells upon stable transfection resuspended in 100 μL DMEM. Subsequently, mice were exposed in 4 Gy radiation after the tumor volumes reaching approximately 300 mm^3^. Tumor volumes and mice body weight were detected every 3 days for 21 days. Mice were sacrificed at 21 days and the tumor weight was evaluated. The expressions of NEAT1 and miR-27b-3p were determined using qRT-PCR.

### Statistical analysis

All data are expressed as means ± standard deviation (SD) from three separate experiments. All statistical analyses were evaluated by SPSS 18.0 statistical software (SPSS, Chicago, IL, USA) with the Student’s *t* test or one-way analysis of variance. A value of *P* less than 0.05 was considered be statistically significant.

## Results

### The expression of NEAT1 was increased in GC tissues and was associated with radiation resistance

**Fig. 1:**
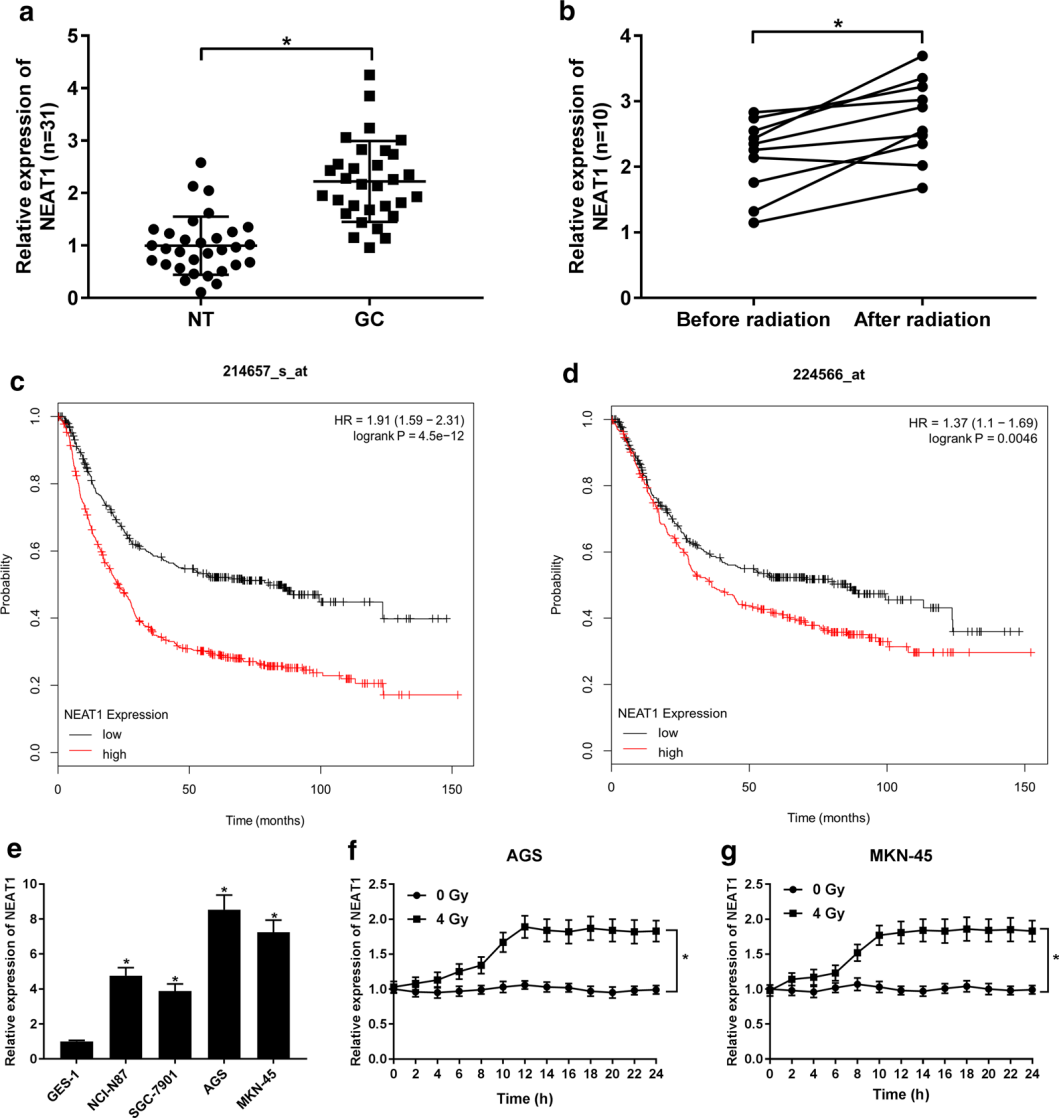
The expression of NEAT1 in gastric cancer tissues. **a** qRT-PCR was performed to examine the expression of NEAT1 in gastric cancer (GC) tissues and normal tissues (NT). **b** qRT-PCR was performed to examine the expression of NEAT1 after the GC patients received radiation treatment. **c**, **d** Kaplan–Meier survival curves for NEAT1 expression in GC, which was created by using KM Plotter (Affymetrix ID: 214,657-s-at and 224,566-at). **e** The expression of NEAT1 in GC cell line (NCI-N87, SGC-7901, AGS and MKN-45) and human gastric epithelium cell line GES-1. **f**, **g** The expression of NEAT1 in AGS and MKN-45 cells treated with 4 Gy radiation for 0 to 24 h. *P < 0.05

**Fig. 2:**
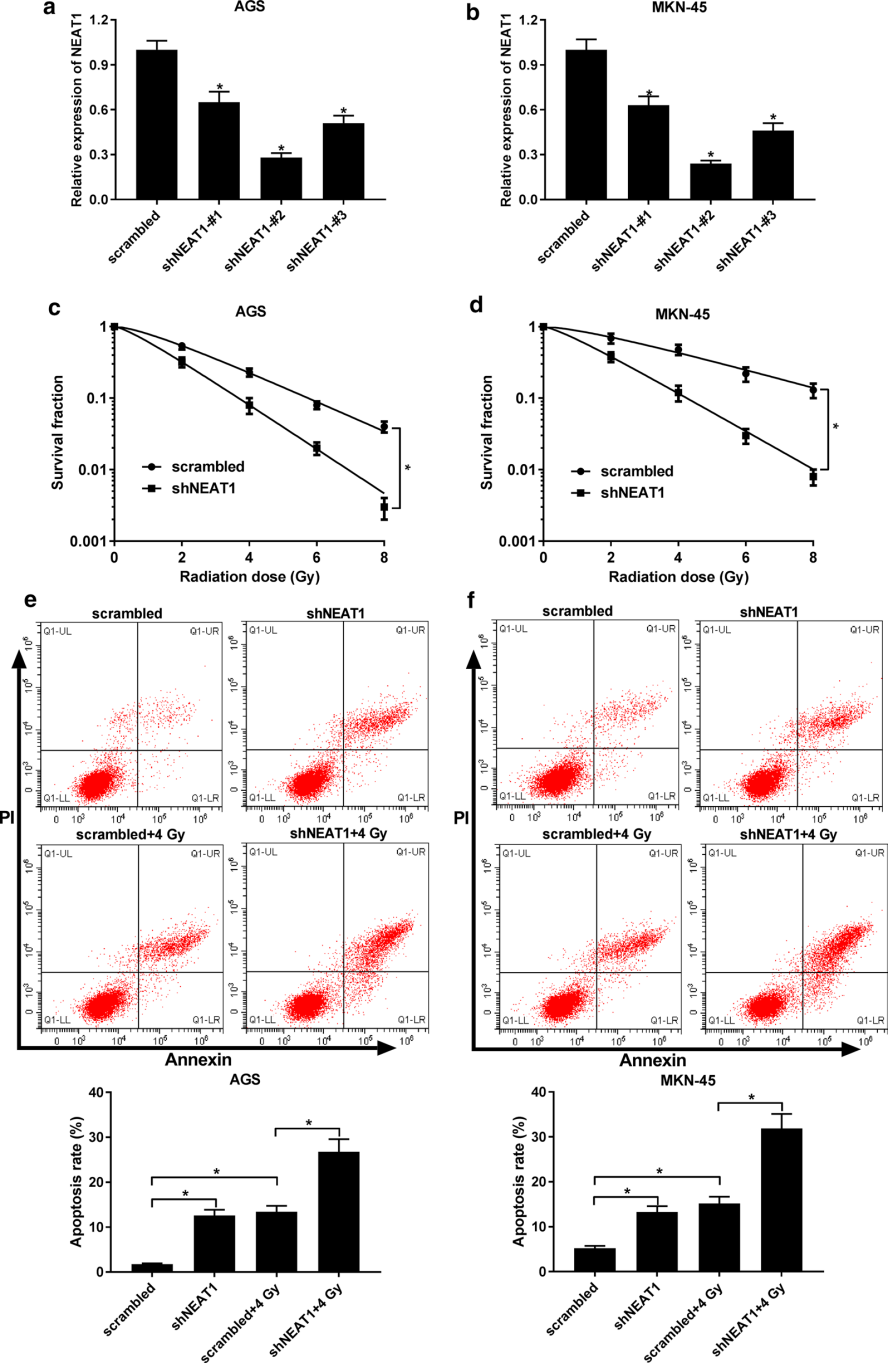
Effect of NEAT1 knockdown on radio-sensitivity of GC cells. **a**, **b** The expression of NEAT1 in MKN-45 and AGS cells transfected with shNEAT1#1, shNEAT1#2, shNEAT1#3, or scrambled. **c**, **d** The survival fraction was determined in scrambled or shNEAT1#2 transfected MKN-45 and AGS cells upon radiotherapy. **e**, **f** Cell apoptosis was examined in radiation-treated MKN-45 and AGS cells transfected with scrambled or shNEAT1#2. *P < 0.05

To examine whether NEAT1 participates in the regulation of GC radiosensitivity, the expression of NEAT1 in 31 GC patients was detected. The result of qRT-PCR demonstrated that NEAT1 was elevated in GC tissues in construct with NT group (Fig. 1a). Besides, another 10 GC patients were recruited to examine the change of NEAT1 expression when patients received IR radiation therapy. As shown in Fig. 1b, the expression of NEAT1 was obviously rising in patients after radiation therapy. Additionally, in a full study GC database, the Kaplan–Meier analysis (www.kmplot.com) presented that GC patients with high expression of NEAT1 had shorter overall survival rate compared with patients with low expression of NEAT1 (Fig. 1c, d). We also evaluated the expression of NEAT1 in Human gastric epithelium cell GES-1 and GC cell lines (NCI-N87, SGC-7901, MKN-45, and AGS). Elevated expression of NEAT1 were detected in different GC cell lines compared with normal gastric cells (Fig. 1e), among them NEAT1 expression in AGS and MKN-45 cells showed the most visible change. Then the expression of NEAT1 in AGS and MKN-45 cells after 4 Gy radiation treatment in 0–24 h was detected. NEAT1 expression was enhanced with the increasing time of treatment and reached the peak at 12 h, and remained stable in the 12 h that follow (Fig. 2f, g).

### Silencing of NEAT1 enhances radio-sensitivity of GC

Given that NEAT1 expression was improved in GC cells after radiation treatment, we thought that NEAT1 might be involved in the radio-sensitivity of GC cells. The loss-of-function experiment was conducted to explore the function of NEAT1 in modulating sensitivity of GC cells to radiation. The transfection efficiency indicated that transfection of shNEAT1#1, shNEAT1#2, and shNEAT1#3 (especially shNEAT1#2) resulted in the reduction of NEAT1 in MKN-45, and AGS cells (Fig. 2a, b). Therefore, we used shNEAT1#2 to downregulated the expression of NEAT1 in the following experiments. Meanwhile, colony formation assay was performed and the shNEAT1-transfected cells were then exposed in 4 Gy radiation for 0 to 8 h. Compared with cells with scrambled transfection, the survival fraction of cells with shNEAT1 transfection was significantly reduced (Fig. 2c, d). Besides, flow cytometry assay also suggested that the depletion of NEAT1 enhanced radiation induced cell apoptosis (Fig. 2e, f).

### NEAT1 negatively regulates the expression of miR-27b-3p

**Fig. 3:**
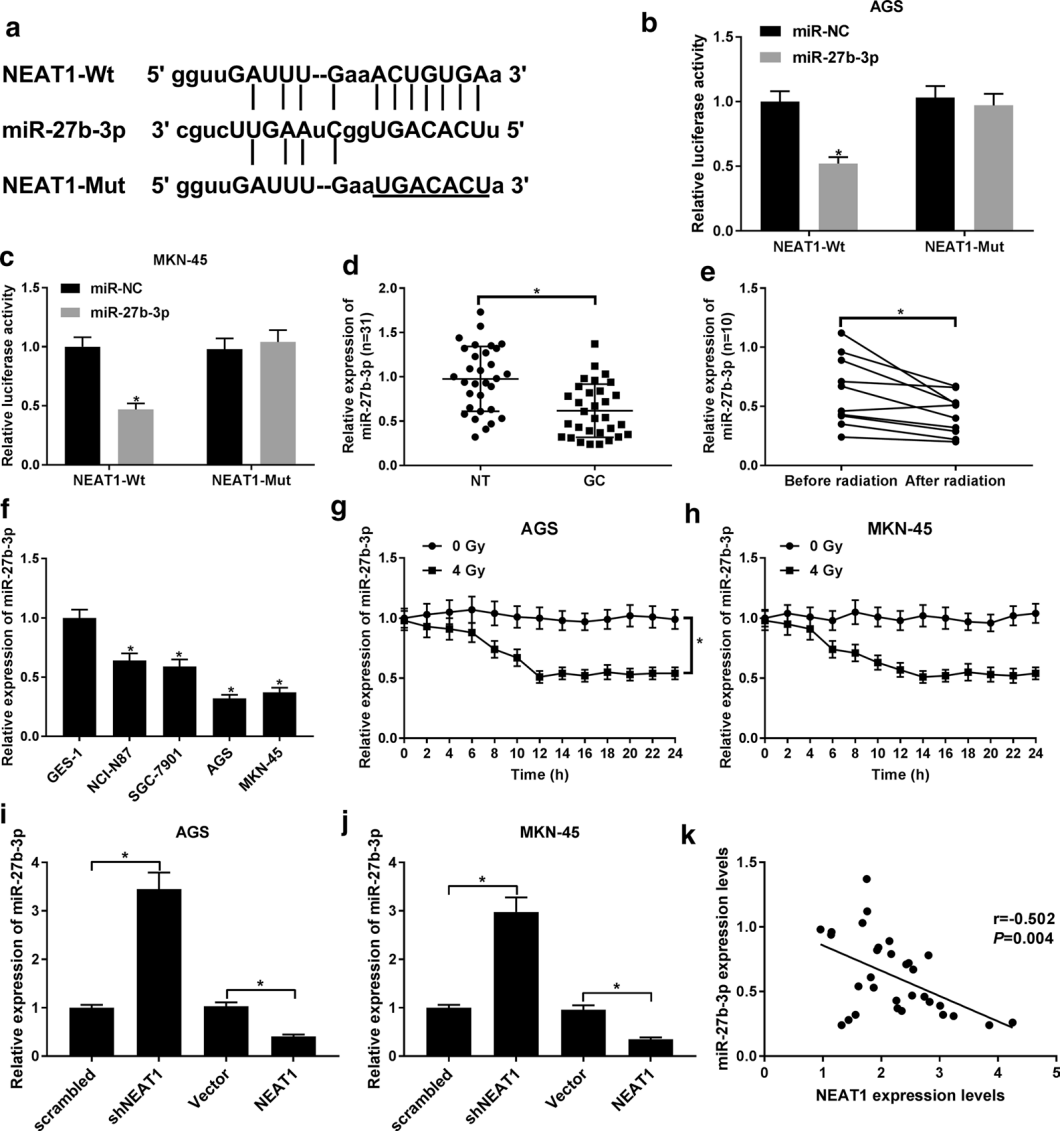
NEAT1 was interacted with miR-27b-3p. **a** The predicted binding sites or mutant binding sites of NEAT1 in miR-27b-3p. **b**, **c** Luciferase reporter assay was carried out to confirm the relationship between miR-27b-3p and NEAT1. **d** qRT-PCR was performed to examine the expression of miR-27b-3p in GC tissues and normal tissues. **b** qRT-PCR was performed to examine the expression of miR-27b-3p after the GC patients received radiation treatment. **f** The expressions of miR-27b-3p in human gastric epithelium cell GES-1 and gastric cancer cell lines (NCI-N87, SGC-7901, MKN-45, and AGS). **g**, **h** The expression of miR-27b-3p in AGS and MKN-45 cells treated with 4 Gy radiation for 0 to 24 h. **i**, **j** The expression of miR-27b-3p in cells transfected with scrambled, shNEAT1, pcDNA vector, or NEAT1 overexpression plasmid. **k** The expression correlation between NEAT1 and miR-27b-3p in GC tissues. *P < 0.05

In consideration of lncRNAs act as a miRNA sponge to participate in the multiple biological process of cancers, we further confirmed whether miRNA participate in the regulation of radio-sensitivity of GC. By using StarBase software, miR-27b-3p was predicted that might combine with NEAT1 (Fig. 3a). The decreasing luciferase activity in cells co-transfected with miR-27b-3p and NEAT1-Wt and the little changed in cells co-transfected with miR-27b-3p and NEAT1-Mut plasmid confirmed that miR-27b-3p was a target of NEAT1 (Fig. 3b, c). Different from NEAT1, miR-27b-3p was downregulated in GC tissues and was further declined when GC patients received radiation therapy (Fig. 3d, e). Besides, compared with GES-1 cells, the expression of miR-27b-3p in GC cell lines (NCI-N87, SGC-7901, MKN-45, and AGS) was also declined (Fig. 3F). Besides, in radiation treatment AGS and MKN-45 cells, miR-27b-3p expression was reduced with the increased time of radiation treatment (Fig. 3g, h). Additionally, shRNA-mediated knockdown of NEAT1 led to a great increase of miR-27b-3p expression (Fig. 3i, j). However, when we overexpressed the expression of NEAT1 in MKN-45, and AGS cells, a remarkable reduction of miR-27b-3p was also observed (Fig. 3i, j). Pearson correlation analysis of the expression of NEAT1 and miR-37b-3p disclosed the negative relationship of NEAT1 and miR-37b-3p (Fig. 3k). Thus, NEAT1 appears to suppress the expression of miR-27b-3p by interacting with it.

### Upregulation of miR-27b-3p increases radio-sensitivity of GC

**Fig. 4:**
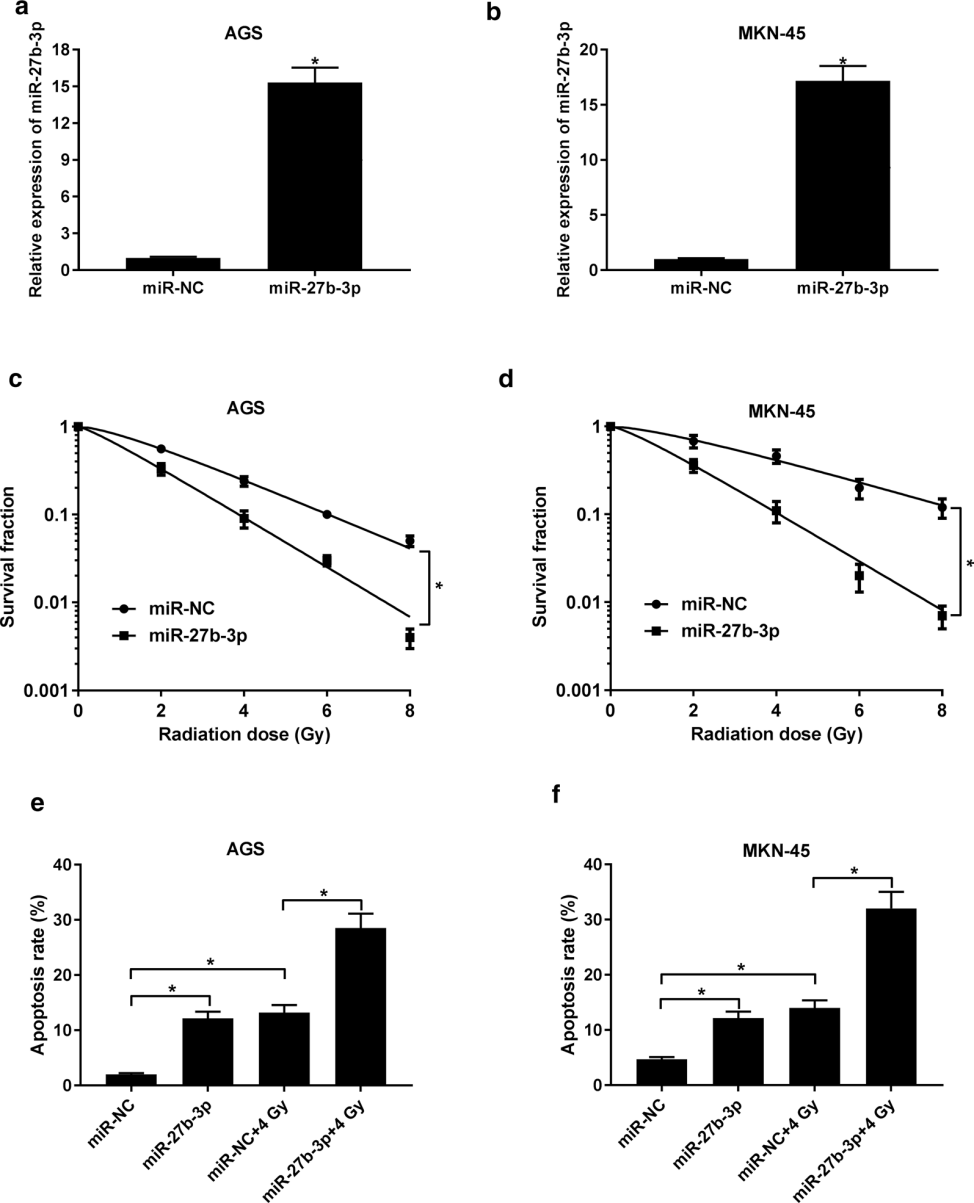
Effect of miR-27b-3p upregulation on radio-sensitivity of GC cells. **a**, **b** The expression of miR-27b-3p in MKN-45 and AGS cells transfected with miR-27b-3p mimic or miR-NC. **c**, **d** The survival fraction was determined in MKN-45 and AGS cells introduced with miR-27b-3p mimic or miR-NC. **e**, **f** Cell apoptosis was examined in radiation-treated MKN-45 and AGS cells. *P < 0.05

To further address the role of miR-27b-3p in regulating radio-sensitivity of GC, we studied the effect of miR-27b-3p overexpression on MKN-45, and AGS cells in response to radiation. As displayed in Fig. 4a, b, the introduction of miR-27b-3p mimic substantially increased the level of miR-27b-3p in cells in construct with miR-NC group. After radiation treatment, we found that elevated expression of miR-27b-3p also decreased the survival fraction (Fig. 4c, d). Moreover, transfection of miR-27b-3p mimic increased radiation-induced apoptosis (Fig. 4e, f). Those data suggested that combination of miR-27b-3p upregulation and radiation therapy may contribute to the increase of radio-sensitivity of GC.

### Downregulation of miR-27b-3p reversed the NEAT1 knockdown-mediated radio-sensitivity of GC cells in vitro and in vivo

**Fig. 5:**
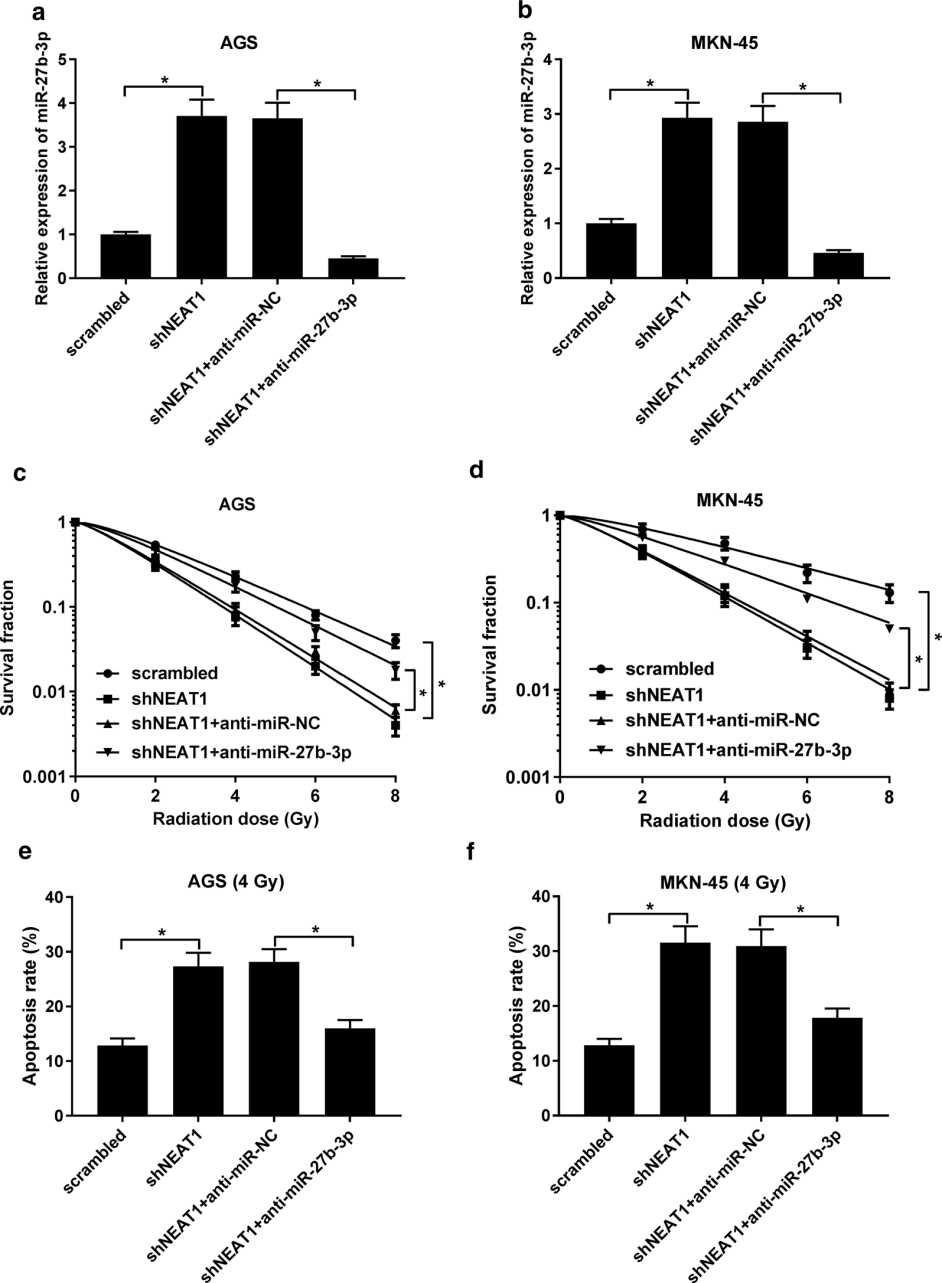
The effect of miR-27b-3p downregulation on the NEAT1 depletion-mediated radio-sensitivity of GC cells in vitro. **a**, **b** The expression of miR-27b-3p in MKN-45 and AGS cells transfected with scrambled, shNEAT1, shNEAT1 + anti-miR-NC, or shNEAT1 + anti-miR-27b-3p. **c**, **d** The survival fraction was determined by MTT assay. (E and F) Cell apoptosis was evaluated by flow cytometry. *P < 0.05

**Fig. 6:**
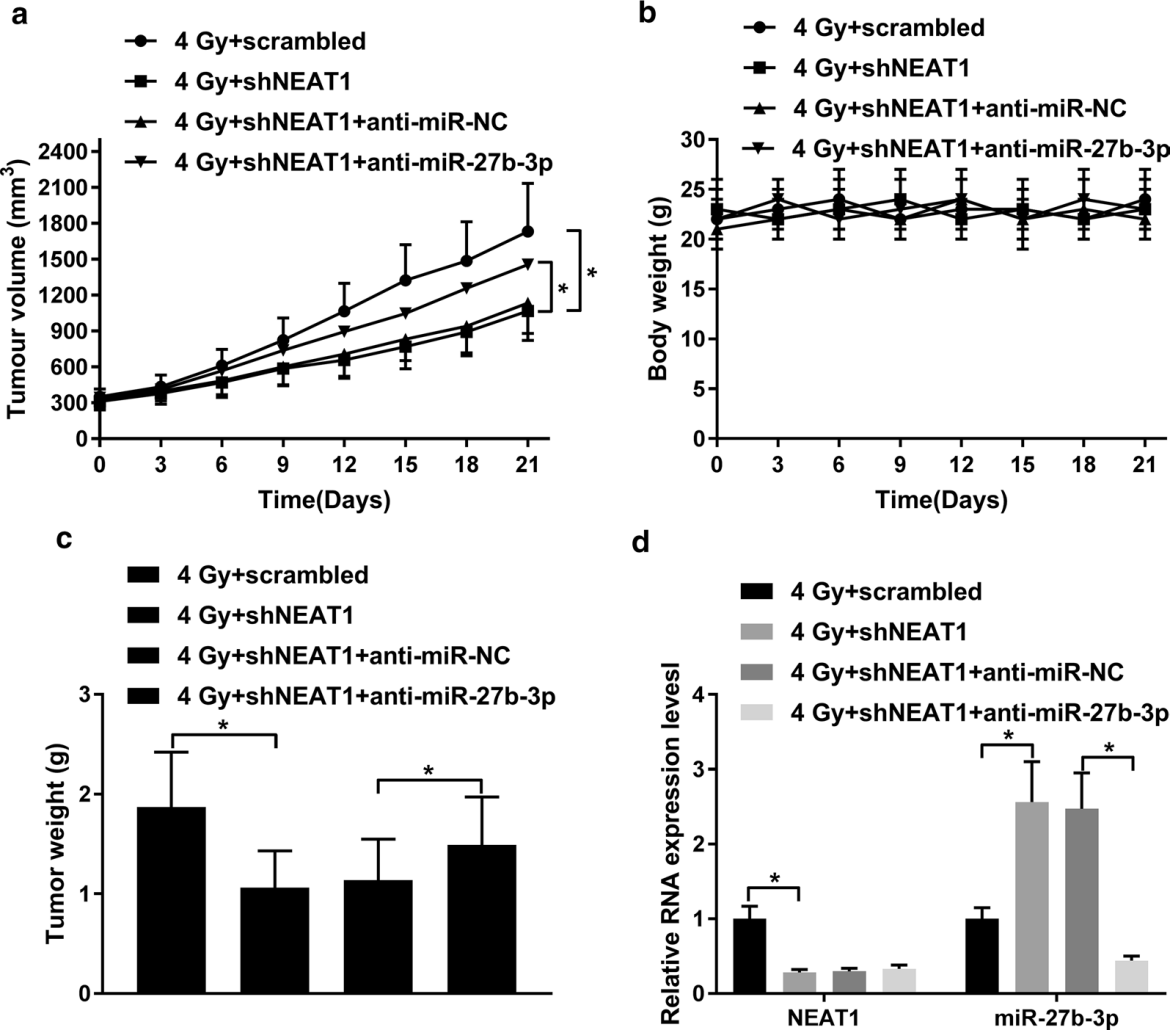
Knockdown of NEAT1 sensitized GC cells to radiotherapy by sponging miR-27b-3p in vivo. MKN-45 cells stably transfected with shNEAT1, scrambled, shNEAT1 + anti-miR-NC, or shNEAT1 + anti-miR-27b-3p were subcutaneously inoculated into nude mice, which received 4 Gy radiation treatment a week. 21 days upon radiation treatment, the tumors were resected and weighted. Tumor volumes (**a**) and mice body weight (**b**) were measured every 3 days after xenograft tumor was irradiated with 4 Gy X-rays. **c** Tumors were weighted at 21 days of radiation. **d** qRT-PCR was conducted to examine the expression of NEAT1 and miR-27b-3p in tumor tissues of MKN-45 cells implanted into nude mice. *P < 0.05

Finally, we further investigate whether NEAT1 regulates radio-sensitivity of GC by sponging miR-27b-3p. Anti-miR-27b-3p and shNEAT1 were co-transfected into MKN-45, and AGS cells. As demonstrated in Fig. 5a, b, the transfection of anti-miR-27b-3p undermined the shNEAT1-mediated promotion of miR-27b-3p expression. Furthermore, the survival fraction was decreased in shNEAT1 + anti-miR-NC group, which was significantly abated by silencing of miR-27b-3p (Fig. 5c, d). Similarly, our results also showed that interference of miR-27b-3p drastically weakened the effect of NEAT1 knockdown on apoptosis (Fig. 5e, f). Hence, we hypothesized that NEAT1/miR-27b-3p axis may be responsible for the radio-sensitivity of GC. Additionally, we further addressed the involvement of NEAT1/miR-27b-3p axis in GC radio-sensitivity in vivo. MKN-45 cells stably transfected shNEAT1, scrambled, shNEAT1 + anti-miR-NC, or shNEAT1 + anti-miR-27b-3p were transfected into the nude mice. Consistently, the in vivo results disclosed that depletion of and miR-27b-3p and NEAT1 presented no obvious change in body weight in relative to other groups (Fig. 6b). However, the combination of shNEAT1 and anti-miR-27b-3p induced a significant increase of tumor volume and weight when compared with that of 4 Gy + shNEAT1 + anti-miR-NC group (Fig. 6a, c). Additionally, further analysis of the expression of miR-27b-3p and NEAT1 indicated that the expression of NEAT1 has no significant change in NEAT1 knockdown alone group or both NEAT1 and miR-27b-3p knockdown group when compared with 4 Gy+ scrambled group. Surprisingly, loss of NEAT1 markedly inhibited the level of miR-27b-3p, which was abated by anti-miR-27b-3p (Fig. 6d). These data confirmed the hypothesis that NEAT1 enhanced radio-sensitivity of GC cells by negatively regulating miR-27b-3p.

## Discussion

Radiotherapy is an important clinical treatment for the majority of GC [16]. However, radio-resistance is a main obstacle of therapeutic failure and recurrence of cancer. Therefore, a deep understand of molecular mechanisms of radio-resistance of GC may contribute to the therapeutic effect and progress of GC patients.

NEAT1 has been reported to be involved in the development of many cancers [17, 18]. For example, NEAT1 is implicated in tumor recurrence and chemotherapy resistance via inducing cancer stem cells (CSC)-like properties in non-small cell lung cancer (NSCLC) cells [19]. In ovarian cancer, NEAT1 is elevated and increases paclitaxel resistance of ovarian cancer via upregulating zinc finger E-box-binding homeobox 1 (ZEB1) expression by sponging miR-194 [18]. Moreover, NEAT1 upregulation promotes proliferation while blocks cell apoptosis by miR-34a-5p/Bcl2 axis [20]. In another report, NEAT1 is upregulated in breast cancer and Kaplan–Meier analysis indicated that high expression of NEAT1 is related to poor overall survival [21]. Peng et al. [22] also revealed that high expression of NEAT1 in colorectal cancer results in adverse survival using Kaplan–Meier analysis. They further demonstrated that silencing of NEAT1 represses cell viability and enhances cell apoptosis. Those data implied that NEAT1 may be promising biomarker for prognosis and the target of treatment for cancers. Recently, Fu et al. [14] also noted that increasing NEAT1 level in GC tumors and cells is associated with clinical stage, histological type, lymph node metastasis, distant metastasis, and poor survival. They further performed MTT, colony formation, and transwell assays and the results disclosed that decrease of NEAT1 suppresses cell growth, migration and invasion. Consistently, previous study also manifested the involvement of miR-335-5p/Rho Associated Coiled-Coil Containing Protein Kinase 1 (ROCK1) axis in the NEAT1-medated function in GC [23]. Moreover, NEAT1 could participate in chemotherapy resistance of GC cells to Adriamycin [24]. However, the investigation about NEAT1 in modulating radio-sensitivity of GC is still on the brink of realization. In line with previous report, our data demonstrated that NEAT1 was highly expressed in GC tissues and cells and was related to poor survival. Besides, we further uncovered that NEAT1 was elevated after radiation treatment in GC. In a word, the siRNA-mediated knockdown of NEAT1 may play a promotion role in X-radiation-mediated radio-sensitivity in GC.

Extensive research has revealed the lncRNA-miRNA interaction in the regulation of radio-sensitivity of tumors [25–27]. miR-27b-3p has been reported to be downregulated in acute kidney injury (AKI) and LINC00520 promotes the development of AKI by modulating miR-27b-3p/Oncostatin M Receptor β (OSMR) axis through the PI3K/AKT pathway [28]. Dong et al. also showed that miR-27b-3p is correlated with the lnc KCNQ1OT1-mediated proliferation and metastasis in NSCLC [29]. A study also revealed the low expression of miR-27b-3p in GC tissues [15]. However, whether miR-27b-3p is required for the radio-sensitivity of GC remain largely elusive. In our research, miR-27b-3p expressed at low level in GC tissues and cells. Contrary to the NEAT1, miR-27b-3p expression was downregulated in tissues and cells exposed to radiation. Meanwhile, upregulation of miR-27b-3p also enhanced radio-sensitivity of GC. More importantly, NEAT1/miR-27b-3p axis has been confirmed in diabetic nephropathy [30]. We further found that NEAT1 could negatively modulated the expression of miR-27b-3p in GC, suggesting that miR-27b-3p may be responsible for the NEAT1-mdeiated radiation sensitivity of GC. Of note, we performed the rescue-of-function experiment in vivo and in vitro. The results fueled that the repression of miR-27b-3p could partly overturn the effect of NEAT1 downregulation on radiation sensitivity in GC. At the same time, NEAT1/miR-27b-3p axis had an implication in the radiotherapy in mice. Collectively, our data implied that NEAT1 depletion enhanced radio-sensitivity of GC through inhibiting survival fraction and promoting apoptosis by targeting miR-27b-3p.

However, our study also has some limitations. We only explored the role of NEAT1/miR-27b-3p axis in radio-sensitivity of GC. The potential downstream target gene of miR-27b-3p as well as underlying signaling pathways remain need a focused in depth exploration.

## Conclusion

In conclusion, our study for the first time provided the evidence that NEAT1 was responsible for radio-sensitivity of GC by sponging miR-27b-3p. NEAT1 might be promising candidate to improve radio-therapy in GC patients.

## Supplementary information

**Additional file 1: Table S1** shRNA, miRNA mimic and miRNA inhibitor sequences.

## Data Availability

All data generated or analysed during this study are included in this published article.

## References

[1] Song Z, Wu Y, Yang J, Yang D, Fang X (2017). Progress in the treatment of advanced gastric cancer. Tumour Biol.

[2] Hamashima C (2014). Current issues and future perspectives of gastric cancer screening. World J Gastroenterol.

[3] Ang TL, Fock KM (2014). Clinical epidemiology of gastric cancer. Singapore Med J.

[4] Karimi P, Islami F, Anandasabapathy S, Freedman ND, Kamangar F (2014). Gastric cancer: descriptive epidemiology, risk factors, screening, and prevention. Cancer Epidemiol Biomark Prev.

[5] Ji F, Sha H, Meng F, Zhu A, Ding N, Zhang H, Xu H, Qian H, Yu L, Liu Q (2018). Tumorpenetrating peptide fused EGFR singledomain antibody enhances radiation responses following EGFR inhibition in gastric cancer. Oncol Rep.

[6] O'Leary BR, Houwen FK, Johnson CL, Allen BG, Mezhir JJ, Berg DJ, Cullen JJ, Spitz DR (2018). Pharmacological ascorbate as an adjuvant for enhancing radiation-chemotherapy responses in gastric adenocarcinoma. Radiat Res.

[7] Renganathan A, Felley-Bosco E (2017). Long noncoding RNAs in cancer and therapeutic potential. AdvExpMedBiol..

[8] Kong Q, Qiu M (2018). Long noncoding RNA SNHG15 promotes human breast cancer proliferation, migration and invasion by sponging miR-211-3p. Biochem Biophys Res Commun.

[9] Li T, Mo X, Fu L, Xiao B, Guo J (2016). Molecular mechanisms of long noncoding RNAs on gastric cancer. Oncotarget.

[10] Ding J, Li J, Wang H, Tian Y, Xie M, He X, Ji H, Ma Z, Hui B, Wang K (2017). Long noncoding RNA CRNDE promotes colorectal cancer cell proliferation via epigenetically silencing DUSP5/CDKN1A expression. Cell Death Dis.

[11] Nie F, Yu X, Huang M, Wang Y, Xie M, Ma H, Wang Z, De W, Sun M (2017). Long noncoding RNA ZFAS1 promotes gastric cancer cells proliferation by epigenetically repressing KLF2 and NKD2 expression. Oncotarget.

[12] Tan J, Qiu K, Li M, Liang Y (2015). Double-negative feedback loop between long non-coding RNA TUG1 and miR-145 promotes epithelial to mesenchymal transition and radioresistance in human bladder cancer cells. FEBS Lett.

[13] Jin C, Yan B, Lu Q, Lin Y, Ma L (2016). The role of MALAT1/miR-1/slug axis on radioresistance in nasopharyngeal carcinoma. Tumour Biol.

[14] Fu JW, Kong Y, Sun X (2016). Long noncoding RNA NEAT1 is an unfavorable prognostic factor and regulates migration and invasion in gastric cancer. J Cancer Res Clin Oncol.

[15] Tao J, Zhi X, Zhang X, Fu M, Huang H, Fan Y, Guan W, Zou C (2015). miR-27b-3p suppresses cell proliferation through targeting receptor tyrosine kinase like orphan receptor 1 in gastric cancer. J Exp Clin Cancer Res.

[16] Zhou J, Wu X, Li G, Gao X, Zhai M, Chen W, Hu H, Tang Z (2017). Prediction of radiosensitive patients with gastric cancer by developing gene signature. Int J Oncol.

[17] Yu X, Li Z, Zheng H, Chan MT, Wu WK (2017). NEAT1: a novel cancer-related long non-coding RNA. Cell Prolif..

[18] An J, Lv W, Zhang Y (2017). LncRNA NEAT1 contributes to paclitaxel resistance of ovarian cancer cells by regulating ZEB1 expression via miR-194. OncoTargets Ther..

[19] Jiang P, Chen A, Wu X, Zhou M, Ul Haq I, Mariyam Z, Feng Q (2018). NEAT1 acts as an inducer of cancer stem cell-like phenotypes in NSCLC by inhibiting EGCG-upregulated CTR1. J Cell Physiol.

[20] Ding N, Wu H, Tao T, Peng E (2017). NEAT1 regulates cell proliferation and apoptosis of ovarian cancer by miR-34a-5p/BCL2. OncoTargets Ther..

[21] Zhao D, Zhang Y, Wang N, Yu N (2017). NEAT1 negatively regulates miR-218 expression and promotes breast cancer progression. Cancer Biomark.

[22] Peng W, Wang Z, Fan H (2017). LncRNA NEAT1 impacts cell proliferation and apoptosis of colorectal cancer via regulation of Akt signaling. Pathol Oncol Res.

[23] Wang H, Zhang M, Sun G (2018). Long non-coding RNA NEAT1 regulates the proliferation, migration and invasion of gastric cancer cells via targeting miR-335-5p/ROCK1 axis. Pharmazie.

[24] Zhang J, Zhao B, Chen X, Wang Z, Xu H, Huang B (2018). Silence of long noncoding RNA NEAT1 inhibits malignant biological behaviors and chemotherapy resistance in gastric cancer. Pathol Oncol Res.

[25] Lai Y, Chen Y, Lin Y, Ye L (2018). Down-regulation of LncRNA CCAT1 enhances radiosensitivity via regulating miR-148b in breast cancer. Cell Biol Int.

[26] Hu X, Jiang H, Jiang X (2017). Downregulation of lncRNA ANRIL inhibits proliferation, induces apoptosis, and enhances radiosensitivity in nasopharyngeal carcinoma cells through regulating miR-125a. Cancer Biol Ther.

[27] Chen M, Liu P, Chen Y, Chen Z, Shen M, Liu X, Li X, Li A, Lin Y, Yang R (2018). Long noncoding RNA FAM201A mediates the radiosensitivity of esophageal squamous cell cancer by regulating ATM and mTOR expression via miR-101. FrontGenet.

[28] Tian X, Ji Y, Liang Y, Zhang J, Guan L, Wang C (2019). LINC00520 targeting miR-27b-3p regulates OSMR expression level to promote acute kidney injury development through the PI3K/AKT signaling pathway. J Cell Physiol..

[29] Dong Z, Yang P, Qiu X, Liang S, Guan B, Yang H, Li F, Sun L, Liu H, Zou G (2019). KCNQ1OT1 facilitates progression of non-small-cell lung carcinoma via modulating miRNA-27b-3p/HSP90AA1 axis. J Cell Physiol.

[30] Wang X, Xu Y, Zhu YC, Wang YK, Li J, Li XY, Ji T, Bai SJ. LncRNA NEAT1 promotes extracellular matrix accumulation and epithelial-to-mesenchymal transition by targeting miR-27b-3p and ZEB1 in diabetic nephropathy. J Cell Physiol. 2019;234(8):12926–33. 10.1002/jcp.27959 PMC13483209 30549040

