# Supplementary material for: Long noncoding RNA NEAT1 regulates radio-sensitivity via microRNA-27b-3p in gastric cancer
Source: Cancer Cell Int. 2020 Dec 3;20:581. doi: 10.1186/s12935-020-01655-4 (PMC7716475; doi:10.1186/s12935-020-01655-4)
Supplement: Supplementary file 1 — Additional file 1: Table S1 shRNA, miRNA mimic and miRNA inhibitor sequences. [file 12935_2020_1655_MOESM1_ESM.docx]

**Additional table S1 shRNA, miRNA mimic and miRNA inhibitor** **sequences**

**shRNA** **sequences: (5’-3’)**

shNEAT1#1 UGGUAAUGGUGGAGGAAGAUU

shNEAT1#2 GUGAGAAGUUGCUUAGAAAUU

shNEAT1#3 GUGAGAAGUUGCUUAGAAAUU

scrambled UUCUCCGAACGUGUCACGU

**miR-27b-3p mimic and inhibitor sequences: (5’-3’)**

miR-27b-3p mimic UUCACAGUGGCUAAGUUCUGC

miR-NC UUCUUCGAACGUGUCACGUTT

anti-miR-27b-3p GCAGAACUUAGCCACUGUGAA

anti-miR-NC CAGUACUUUUGUGUAGUACAA
